# Life-Threatening Complications of Hormonal Contraceptives: A Case History

**DOI:** 10.1155/2013/186230

**Published:** 2013-05-20

**Authors:** Saheed Khan, Yvo M. Smulders, Johanna I. P. de Vries, Angélique M. E. Spoelstra-de Man

**Affiliations:** ^1^Department of Intensive Care, VU Medical Center, Postbus 7057, 1007 MB Amsterdam, The Netherlands; ^2^Department of Internal Medicine, VU Medical Center, Postbus 7057, 1007 MB Amsterdam, The Netherlands; ^3^Department of Gynaecology, VU Medical Center, Postbus 7057, 1007 MB Amsterdam, The Netherlands

## Abstract

We present a case with the rare combination of thrombotic and hemorrhagic complications of oral contraceptives. A healthy 40-year-old woman suffered from cardiac arrest due to massive pulmonary embolism, caused by oral contraceptives and immobilization during a flight. After successful resuscitation, obstructive shock necessitated thrombolysis and thereafter heparin. Anticoagulation was complicated by internal bleeding from contraceptive related hepatic adenoma. She underwent arterial embolisation, and anticoagulation was continued. On day 18, she was discharged in a good condition. Hepatic adenomas are a potential source of internal bleeding in women using oral contraceptives requiring anticoagulation. Signs of internal bleeding in such patients should prompt immediate abdominal ultrasound examination.

## 1. Introduction

Oral contraceptives (OAC) are used worldwide and generally considered safe. Severe adverse events are rare and usually related to thromboembolism and neoplasm. The dose of the estrogen constituent and the type of progestin determine the risk of venous thromboembolism [1]. Another, less well-known complication is hepatic adenoma, which is related to the use of oral estrogens. We present a case with these two life-threatening complications of OAC at the same time, which confronted us with a therapeutic challenge.

## 2. Case

A 40-year-old woman travelled by plane from Tanzania to the UK. She had an unremarkable medical history (*G*_0_ *P*_0_) and only used a fourth generation OAC (Yasmin: ethinylestradiol/drospirenone). During a stopover in Amsterdam, she collapsed and was found to be pulseless. Basic life support was started immediately and when the paramedics arrived, she had pulseless electrical activity. After 55 minutes of resuscitation, spontaneous circulation returned. Upon arrival in our hospital, physical examination showed sinus tachycardia 150/min, blood pressure 90/60 mmHg with norepinephrine 0.4 mcg/kg/min, central venous pressure 22 mmHg, and oxygen saturation 92%. Relevant results of the blood examination are shown in Table 1. 

After initial stabilisation, chest CT angiography revealed extensive bilateral pulmonary embolism. Despite the coagulation disorders, presumed to be due to disseminated intravascular coagulation, we administered recombinant tissue plasminogen activator (r-tPA), followed by heparin, because of persistent obstructive shock. Within a few hours, her hemodynamic condition improved. She was also treated with therapeutic hypothermia. After cessation of the sedation on day 3, she gradually recovered from postanoxic encephalopathy. 

**Figure 1:**
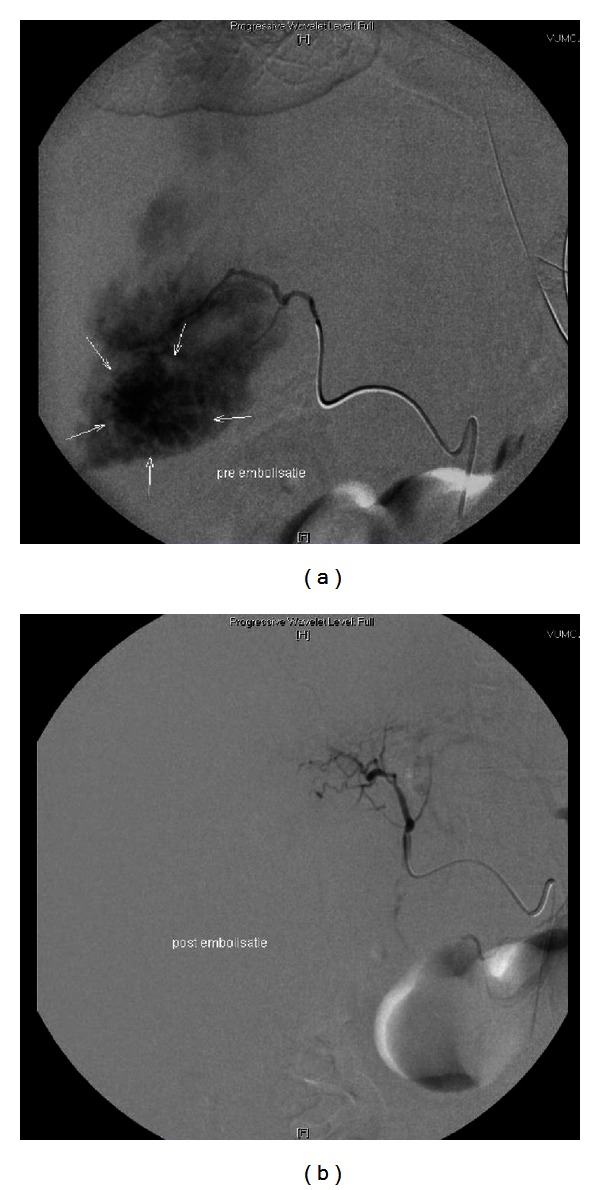
(a) Angiography before embolization; arrows depict the area with bleeding. (b) Angiography after embolization.

On the first 3 days, her haemoglobin level declined significantly, requiring 7 units of erythrocyte concentrates to maintain a haemoglobin level above 8 g/dL. There was no external source of blood loss. Bedsides, ultrasound on day 3 showed free fluid around the liver, and abdominal CT angiography revealed ongoing bleeding in a 4 × 4 × 4 cm large hepatic adenoma. Conventional angiography with embolisation of the left hepatic artery was performed successfully (Figure 1). Over the next days, she had no signs of rebleeding, and heparin was continued throughout the whole event. On day 7, she was successfully extubated and thereafter transferred to the general ward in a good clinical condition with a maximal Glasgow Coma Score. On day 18, she was discharged from the hospital. 2.5 years later, she is still in good health. 

## 3. Discussion

Our patient suffered from 2 life-threatening complications of OAC use, that is, massive pulmonary embolism resulting in cardiac arrest and contraceptive related hepatic adenoma with hemorrhage after thrombolysis. 

Immediately after admission, the laboratory results of our patient were suggestive for disseminated intravascular coagulation (DIC). Both DIC and prolonged resuscitation are relative contraindications for thrombolysis. However, because of the hemodynamic instability, we decided to administer r-tPA. After thrombolysis, our patient required multiple blood transfusions without a clear bleeding focus. Just on the third day, we diagnosed a major hepatic hemorrhage while the patient was on heparin. Because of the recent severe pulmonary embolism, we chose to continue the heparin infusion and performed angiography to identify the bleeding focus, which was successfully embolised. 

The risk of a thrombotic event is 9-10 per 10.000 women/year using hormonal contraception, as compared to 5 per 10,000 women/year in nonusers [1]. Despite doubling of the relative risk, the absolute risk remains low. The dose of the estrogen constituent and the type of progestin determine the risk of venous thromboembolism [1]. Second generation preparations contain reduced dose of both constituents, and the third/fourth generations contain a different type of progestin [2, 3]. Estrogen results in a procoagulant state because it raises the levels of procoagulant factors II, VII, VIII, and X, and von Willebrand factor induces activated protein C resistance and reduces the anticoagulation protein S and antithrombin [4]. Progestins constituents of the third and fourth generation pills increase the estrogen-induced activated protein C resistance and may be more thrombogenic [1, 2, 5, 6]. Additional thrombotic risk factors have a synergistic effect, for example, long-haul air travel raises thrombosis risk 14-fold in contraceptive users [7]. Women are supposed to be counseled on the thrombotic risk of  hormonal contraceptives and preferred medication [3, 8].

The risk of hepatic adenomas is 30-fold higher in women using OAC. The estimated incidence is 3 to 4 per 100,000 women per year, related to the dose and duration of  hormonal contraceptive usage [9]. The estrogen component of OAC may cause transformation of hepatocytes via binding to steroid receptors [10] and inhibition of collagen biosynthesis, affecting the architecture of the vascular wall. This leads to large adenomas (up to 30 cm), which are well vascularised with thin-walled blood vessels, prone to rupture [11–14]. Rates of spontaneous bleeding are 20–40%, and the risk is probably increased during anticoagulation [15]. The standard treatment is emergency laparotomy with gauze packing or partial liver resection to achieve adequate homeostasis. However, this is a major surgical procedure with considerable morbidity and mortality [16, 17]. In recent years, selective arterial embolisation has increasingly been applied as initial treatment. It appears to be a safe and effective method to achieve hemostasis in bleeding hepatic adenoma [16, 17] and even allowed us to continue heparinisation in our patient.

The combination of a simultaneous thrombosis and a bleeding hepatic adenoma due to OAC used immediately after initiation of anticoagulation may be fatal to young patients. To the best of our knowledge, this has only been reported once before in 1977 [18]. Hepatic adenomas occur more frequently in women using OAC; therefore in patients using OAC who require anticoagulation, we recommend considering hepatic adenoma in case of signs of internal bleeding. 

## 4. Conclusion

In this case, the rare simultaneous combination of thrombotic and hemorrhagic complications of OAC was effectively treated with selective arterial embolisation during ongoing heparinisation. Abdominal ultrasound examination can be considered in patients using OAC who require anticoagulation to be timely aware of hepatic adenomas with the potential of severe bleeding. 

## Figures and Tables

**Table 1:** Laboratory results on admission in hospital.

pH	6.62	(7.35–7.45)
*p*CO_2_ (mmHg)	87	(35–45)
*p*O_2_ (mmHg)	126	(>80)
Bicarbonate (mmol/L)	8	(22–28)
Base excess	−13	(−3–+3)
O_2_ saturation (%)	98	(>90%)
Lactate (mmol/L)	17	(<2.2)
Hemoglobin (g/dL)	10.5	(12–17)
Thrombocytes (10^9^/L)	157	(150–400)
Leucocytes (10^9^/L)	12	(4–10)
APTT (sec)	95	(25–40)
Prothrombin time (INR)	1.81	(0.8–1.2)
Fibrinogen (g/L)	0.8	(2–4)
